# In Vitro Technologies for American Chestnut (*Castanea dentata* (Marshall) Borkh) Conservation

**DOI:** 10.3390/plants11030464

**Published:** 2022-02-08

**Authors:** Zhuoya Liu, Wen-Lu Bi, Mukund R. Shukla, Praveen K. Saxena

**Affiliations:** Department of Plant Agriculture, Gosling Research Institute for Plant Preservation, University of Guelph, Guelph, ON N1G 2W1, Canada; joryaliu24@gmail.com (Z.L.); wenlubi@uoguelph.ca (W.-L.B.); mshukla@uoguelph.ca (M.R.S.)

**Keywords:** American chestnut, micropropagation, rocker culture system, cryopreservation, shoot tips, in vitro technologies

## Abstract

American chestnut (*Castanea dentata*), a native species of eastern North America, is an economically important deciduous hardwood tree that has been designated as endangered in Canada. The population of American chestnut trees has dwindled significantly across Southern Ontario due to chestnut blight and many of the surviving trees continue to show blight disease symptoms. American chestnut requires efficient strategies for propagation and preservation for species recovery. The objective of this study was to develop a long-term plant conservation program using micropropagation and cryopreservation protocols. An in vitro technology using a liquid-based temporary immersion system (TIS) was developed for micropropagation of American chestnut. The highest rate of shoot multiplication was observed in cultures grown in the DKW (Driver and Kuniyuki 1984) basal medium supplemented with 2.2 µM 6-benzylaminopurine and 1.0 µM gibberellic acid. More than 95% of proliferated microshoots, about 40–50 mm in size, developed roots after 30 days of culture within bioreactor vessels containing DKW basal medium supplemented with 15 µM 3-Indolebutyric acid. Rooted plantlets transplanted to the greenhouse had a survival efficiency of 82% after one month of growth. The cryopreservation protocol for germplasm preservation was developed through droplet vitrification of shoots. Optimal regeneration of shoot tips occurred from explants precultured on stepwise concentrations of sucrose and subsequent dehydration in PVS3 for 30 min. Cryopreserved shoot tips were regenerated to whole plants using pre-optimized conditions of micropropagation. This study confirms the potential of TIS for micropropagation in ex situ conservation and reintroduction of endangered American chestnuts and possibly other woody plant species.

## 1. Introduction

*Castanea dentata* (Marshall) Borkh., commonly known as American chestnut, is an ancient tree species of great economic, cultural, and ecological importance. Before the 1900s, American chestnut trees were dominant in the eastern forests of North America and highly valued for their naturally rot-resistant wood and precious nuts. The massive footprint of American chestnut trees, estimated at more than four billion, was devastated by the fungal blight pathogen *Cryphomectria parasitica* (Murr.) Barr [1,2,3]. Since then, the giant mature American chestnuts trees have been virtually extinct for several decades. Moreover, urbanization and deforestation for farming and industrial development dramatically reduced the natural habitat of the American chestnut. The Species at Risk in Ontario (SARO) has classified American chestnut as an endangered species which is currently under the protection of the Recovery Strategy in Ontario [4]. There are approximately 2000 extant American chestnut individuals across southern Ontario that need urgent conservation to ensure long-term preservation of this species [3].

Mass propagation and reintroduction in natural habitats is an effective strategy for increasing the declining populations of endangered species [5,6,7]. Traditional propagation systems of the American chestnut are not efficient for large-scale propagation of plants for conservation or breeding programs to develop disease- resistant germplasm [8,9]. In vitro propagation is a highly effective tool for rapid, large-scale cloning of disease-free germplasm from limited plant material in controlled environment conditions, as well as, for long-term maintenance of the germplasm [10]. The American chestnut has been propagated in vitro through proliferation of shoots [11,12,13,14] and somatic embryogenesis [15,16,17,18,19,20,21]. However, these micropropagation protocols produced limited multiplication which is not amenable to large-scale plant production. In addition, micropropagation using a semi-solid medium is resource-intensive, time-consuming, and increases production cost by 10 to 20% [22]. A liquid in vitro culture system enables uniform and continuous accessibility of nutrients to growing explants, which subsequently leads to an improvement in biomass, shoot multiplication, rooting, and whole plant production compared to responses on the semi-solid medium [22,23,24]. The use of a temporary immersion system (TIS) has become an ideal option for micropropagation as it facilitates multiplication of robust plants with uniform physiology [25,26,27]. The plant materials in TIS are cultured in liquid medium with a brief periodic immersion followed by alternate exposure for gas exchange in order to avoid hyperhydricity, which commonly occurs in complete suspension of tissues in the liquid medium [25,28]. The TIS has been successfully used for micropropagation of several woody species such as tea [29], Calabash Tree [30], papaya [31], breadfruit [32], apple root stocks [33], and hazelnut [34,35]. Micropropagation of the chestnut species through TIS has also been reported for the European chestnut (*Castanea sativa*) and hybrid chestnuts (*Castanea sativa* × *C. crenata* or *C. sativa* × *C. mollisima*) [36,37,38].

Cryopreservation is a reliable technique for long-term storage of biological samples at ultra-low temperature in liquid nitrogen (LN, −196 °C). Maintenance of plant genetic resources via cryopreservation is considered as one of the most effective options for preservation of plant germplasms ex situ in a contamination-free environment [39,40]. Plant organs and tissues suspended in liquid nitrogen cease metabolic processes at cryogenic temperatures (−140 °C to −196 °C) and can be stored for an infinite period [41,42,43]. Shoot tips or apical meristems are preferred tissues for cryopreservation of genetic resources due to their genetic stability and capacity to reproduce identical clones of the mother plant [40,41]. The first cryopreservation study of *Castanea* species was on the European chestnut (*Castanea sativa* Mill.), in which shoot tips of five cultivars were successfully cryopreserved by vitrification procedure [44]. The highest shoot recovery rate (36%) was observed for terminal buds, as compared to the axillary buds which showed 8.3% recovery after cryopreservation [45]. A similar study conducted by Vidal et al. [46] for cryopreservation of in vitro grown apical meristems in the European chestnut using the vitrification procedure achieved 40–60% recovery after eight weeks of culture. Droplet-vitrification is a preferred method of cryopreservation due to its cost efficiency and simple operation compared to vitrification [40]. The cryopreservation protocols involving droplet-vitrification have been successfully applied to a large number of diverse plant species including woody plants [47,48,49], crops [47,50,51], ornamentals [47,52,53,54], endangered species [55,56,57,58], and medicinal plants [59]. However, despite significant advancements in droplet-vitrification techniques, an efficient cryopreservation protocol for the American chestnut has not been developed.

The objective of this study was to develop an efficient micropropagation protocol for in vitro propagation of the American chestnut using the temporary immersion system. The liquid-based temporary immersion system was compared with the conventional semi-solid system for assessment of its efficiency for optimum shoot multiplication. Apical meristems of micropropagated shoots were used to develop the cryopreservation method through droplet vitrification for the long-term preservation. The effect of preculture treatments and plant vitrification solutions were also examined for improving regeneration of cryopreserved shoot tips of the American chestnut.

## 2. Results

### 2.1. Micropropagation

#### 2.1.1. Establishment of Shoot Cultures

**Figure 1:**
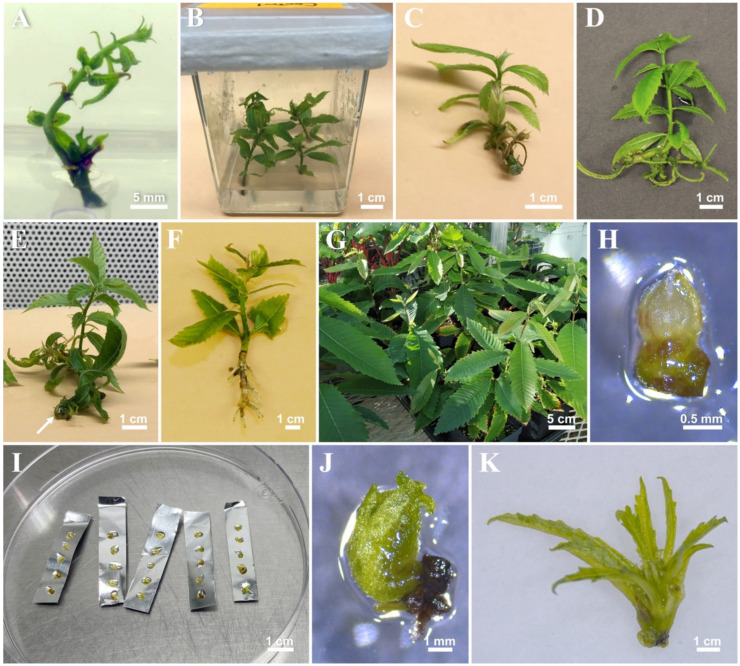
In vitro culture of the American chestnut initiated using shoot buds (**A**); New shoot growth observed on semi-solid DKW basal medium after six weeks (**B**); Shoot proliferation and development after six weeks in the semi-solid culture system (**C**); in the rocker-based liquid temporary immersion system (**D**); and in continuous immersion system (**E**) (The white arrow in E shows the hyperhydricity of leaves); Rooted microshoot obtained after in vitro rooting using OASIS^®^ Rootcubes^®^ in the liquid rooting medium in temporary immersion system (**F**); Acclimatized plants growing in the greenhouse after 12 weeks of transplanting (**G**); A shoot tip taken from in vitro stock cultures (**H**) for cryopreservation using droplet-vitrification method and five shoot tips covered in PVS3 drops placed on aluminum foil strips (**I**); A regenerated shoot tip after four-week post-culture in 2.2 uM 6-benzylaminopurine and 1.0 µM gibberellic acid solution following cryopreservation (**J**); and shoot regrowth observed after eight-week post-culture period following cryopreservation (**K**).

Shoot buds collected from greenhouse-grown plants were cultured successfully in semi-solid DKW basal medium with an average of 57% of the shoot buds remaining free of contamination after 12 days of incubation (Figure 1A,B). Relatively better responses of shoot length and proliferation were observed on the medium with DKW basal salts with vitamins compared to the MS and WPM basal media (Figure S1). 

#### 2.1.2. Comparison of Semi-Solid System (SS), Temporary Immersion System (TIS), and Continuous Immersion System (CIS) for Shoot Multiplication and Growth 

**Figure 2:**
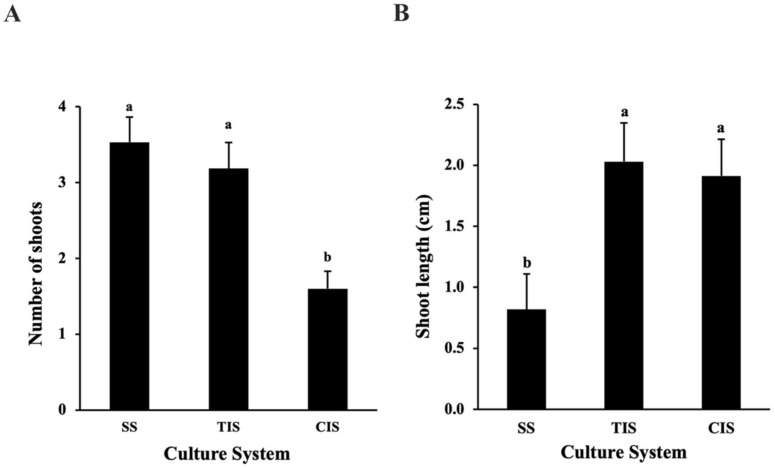
Evaluation of semi-solid culture system (SS), temporary immersion system (TIS), and continuous immersion system (CIS) on shoot multiplication (**A**) and shoot length (**B**) for in vitro shoot multiplication of the American Chestnut. Error bars represent the mean ± standard error. Means followed by the different letter are significantly different according to Tukey-Kramer HSD test (α = 0.05).

A comparison of three culture systems demonstrated that shoot segments cultured in the liquid TIS generated the best shoot growth compared to that observed in other treatments (Figure 1C). The cultures in the SS (3.53 ± 0.33 shoots) and the TIS (3.19 ± 0.34 shoots) generated similar numbers of shoots, which were significantly higher than those observed in the cultures grown in the CIS (1.60 ± 0.23 shoots) (Figure 2A). Shoots grown in the TIS were significantly longer (2.03 ± 0.32 cm) than those in the SS (0.82 ± 0.29 cm) (Figure 2B). Leaves developed on shoots in the liquid medium were larger and greener than those in the SS (Figure 1C). Although the CIS (1.91 ± 0.30 cm) showed similar length of shoots as the TIS (2.03 ± 0.32 cm), the shoots and leaves that remained continuously immersed in the liquid medium in the CIS had much worse symptoms of hyperhydricity in the form of a glassy appearance of the leaves (Figure 1E; white arrow). Therefore, the liquid TIS was considered the most suitable culture system for in vitro growth of American chestnut and used for the rest of the study.

**Figure 3:**
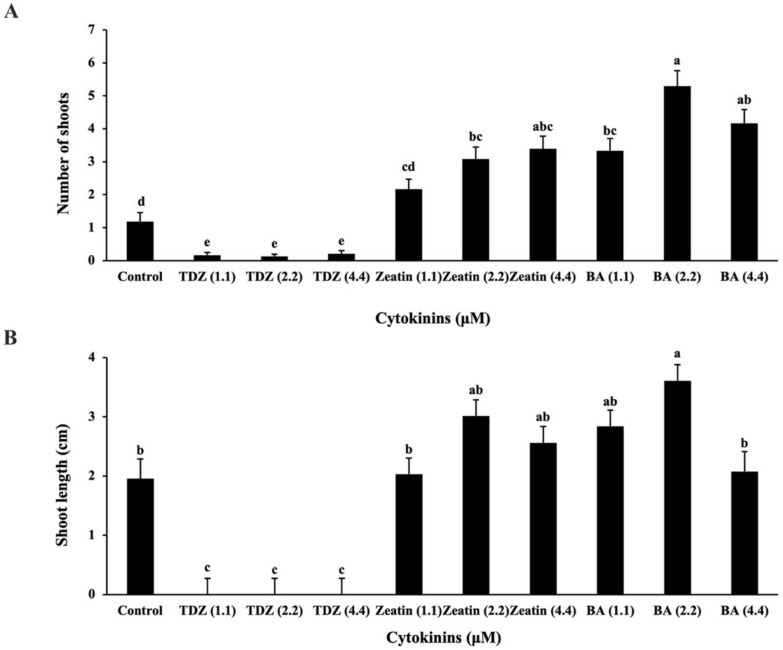
The effect of different cytokinins on numbers of shoot (**A**) and shoot length (**B**). Error bars represent the mean ± standard error. Means followed by the different letter are significantly different according to Tukey–Kramer HSD test (α = 0.05).

A comparison of three commonly used cytokinins (TDZ, BA and Zeatin) showed different effects on shoot multiplication in the liquid TIS. Basal medium supplemented with 2.2 μM BA produced a significantly higher number of shoots (5.29 ± 0.47) and higher shoot length (3.61 ± 0.27 cm) compared to the other treatments (Figure 3). There was no significant difference in shoot length between treatments with the BA at 1.1 μM (2.84 ± 0.27 cm) and 2.2 μM (3.61 ± 0.27 cm) and the Zeatin at 2.2 μM (3.02 ± 0.27 cm) and 4.4 μM (2.56 ± 0.28 cm), while at 2.2 μM BA a higher number of shoots proliferated compared to the Zeatin and the BA at 1.1 μM (Figure 3). Even though the explants cultured in the medium with the BA at 2.2 and 4.4 μM had similar numbers of shoots, shoot elongation was higher in 2.2 μM BA compared to a higher concentration of the BA (4.4 μM). By contrast, cultures treated with the TDZ showed limited formation of shoots (Figure 3) and the shoots tended to grow abnormal leaves close to the apical buds of the explants. The treatment with the BA at 2.2 μM induced healthy shoots and generally produced larger leaves than other treatments. 

The addition of GA_3_ to the culture medium at 1.0 μM promoted a significantly higher shoot length and the number of longer shoots compared to other GA_3_ levels (0.5 and 2 μM) as well as the control (Figure S2).

#### 2.1.3. In Vitro Rooting and Acclimatization

Results of preliminary experiments showed that the shoots cultured in full-strength DKW basal salt mixture containing NAA or IBA at 2.5, 5.0, 10.0 μM failed to develop roots. When grown in half-strength DKW basal medium, 83% of the shoots developed roots and showed a significantly higher number of primary roots (5.73 ± 0.44) on the medium supplemented with 10 μM IBA as compared to other treatments (Figure 1H and Figure S3). 

**Figure 4:**
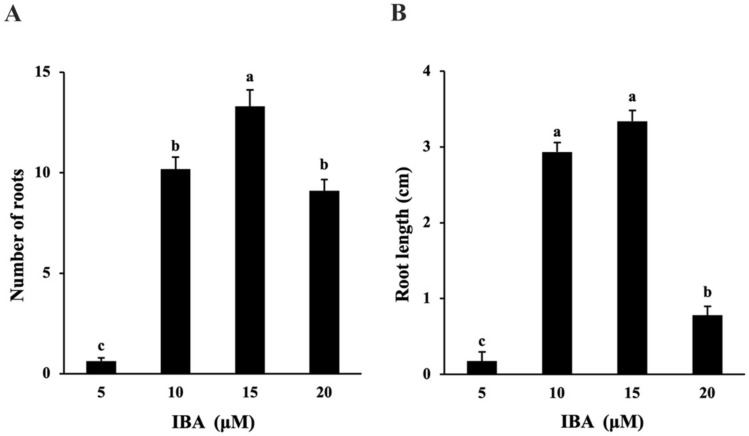
Evaluation of various IBA concentrations (5.0, 10.0, 15.0, and 20 μM) on the number of roots (**A**) and root length (**B**) during in vitro rooting of American chestnut. Error bars represent the mean ± standard error. Means followed by the different letter are significantly different according to Tukey–Kramer HSD test (α = 0.05).

The induction of microshoots with 15 μM IBA in a half-strength DKW basal medium produced 100% rooting response. These shoots showed a significantly higher number of primary roots (13.3 ± 0.82) than those exposed to 10µM IBA (10.18 ± 0.60; Figure 4A). Shoots grown on 10 µM IBA also showed 100% rooting and formed roots of similar length (2.94 ± 0.12 cm) compared to those obtained with 15 µM IBA (3.34 ± 0.14 cm; Figure 4B). Healthy, well-developed, roots were formed within three weeks of culture in the liquid TIS with Rootcubes^®^ (Figure 1H). Initial experiments showed that shoots cultured in semi-solid medium did not form roots until the third week of culture and only 55% of the shoots rooted in the fifth week. Thus, in view of the longer period required for root formation in the semi-solid, the liquid TIS with half-strength DKW basal medium containing 15 μM IBA was considered optimal for root development in vitro.

After two weeks of acclimatization in the mist bed, the plantlets were transplanted into the greenhouse where they showed 82% survival after three months of growth in ex vitro conditions (Figure 1I).

### 2.2. Cryopreservation

Cryopreserved shoot tips turned blackish in color after immersion in LN on post-culture medium but surviving shoot tips turned green after 10 days of culture under normal light and the regenerated meristems continued to grow within four weeks (Figure 1F). Cryopreserved shoot tips which remained brownish after four weeks on post-culture medium were considered dead. Healthy shoots with normal regrowth were green and had elongated leaves (Figure 1G).

#### 2.2.1. Preculture

Preculturing shoot tips in 0.5 M sucrose for 24 h resulted in maximum survival (100%), followed by a decline in survival of shoot tips exposed in 0.5 M sucrose for 48 h (90%), 72 h (80%), and 96 h (71%). None of the shoot meristems survived during preculture on 1.0 M sucrose. Cryopreserved shoot tips (+LN) demonstrated a similar regeneration frequency (20 to 40%) on preculture treatment with 0.5 M sucrose solution for 48–96 h (Table 1). Although 24 h exposure to 0.5 M sucrose supported 86.7% regeneration of non-cryopreserved (−LN) shoot tips, none of the cryopreserved (+LN) shoot tips regenerated after 24 h preculture treatment (Table 1). Shoot tips precultured in a stepwise concentration of sucrose showed the maximum survival (100%) without cryostorage and improved regeneration (55%) of cryopreserved shoot tips compared to those in continuous 0.5 M sucrose (Table 1). A cryopreservation protocol through droplet vitrification was carried out following stepwise preculture of shoot tips in various sucrose concentrations (Figure 1D–G).

#### 2.2.2. Dehydration

**Figure 5:**
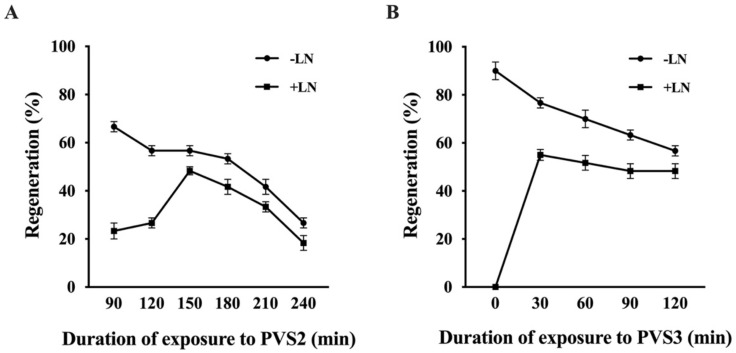
Comparison of different PVS2 (**A**) and PVS3 (**B**) duration on shoot tip cryopreservation using droplet-vitrification method. Regeneration was counted after four weeks. Data is shown as means ± standard errors. Means followed by same letters are not significantly different at *p* = 0.05 according to Tukey–Kramer HSD test.

Cryopreserved shoot tips exposed to PVS2 showed 18.3 to 48.3% regeneration. The regeneration of cryopreserved shoot tips increased from 26.7% (120 min) to significantly higher levels of 48.3% (150 min) and 41.7% (180 min), followed by a decline in longer exposures (Figure 5A). Shoot tips without cryopreservation showed a decreased regeneration with longer duration of PVS2 treatments (Figure 5A).

The time of exposure to PVS3 between 30 to 120 min did not have a significant effect on regeneration of cryopreserved shoot tips (48–55%; Figure 5B). Shoot tips that were not treated with cryoprotective solution for dehydration failed to regenerate after cryopreservation (Figure 5B). Similar results were observed in PVS3 treatments without cryopreservation (−LN) in which regeneration percentage decreased significantly with longer duration (Figure 5B). The highest regeneration (55%) was observed in cryopreserved shoot tips that had been treated with PVS3 for 30 min.

Shoot tips which survived cryopreservation and were post-cultured on filter paper with DKW medium supplemented with 3% sucrose, 2.2 µM BA, and 1.0 µM GA3 showed a slightly higher regrowth of 55% than 48% for those post-cultured on the DKW medium supplemented with 3% sucrose, 2.2 µM BA, and 2.9 µM IAA. Shoot tips from PVS2 and PVS3 without cryopreservation survived with 88% and 87% regrowth, respectively, on the post culture media (data not shown). 

#### 2.2.3. Differential Scanning Calorimetry Analysis

**Figure 6:**
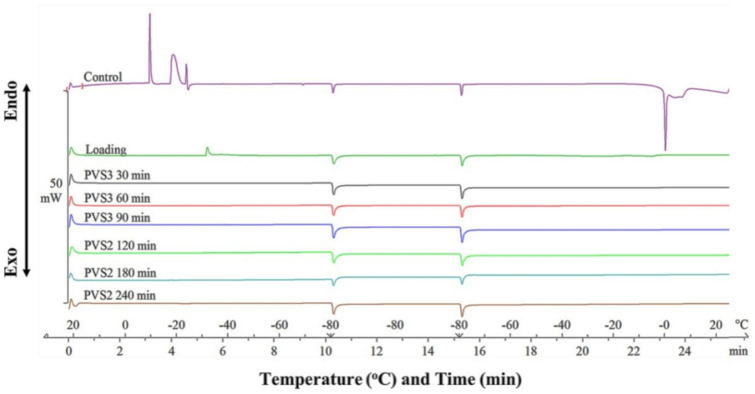
Combined Differential Scanning Calorimetry (DSC) thermograms for comparison of different PVS2 and PVS3 duration on shoot tip cryopreservation as compared to the control and loading solution. Samples were held in DSC at 20 °C, followed by cooling to −80 °C with a cooling rate of −10 °C min^−1^, and kept isothermally for 5 min prior to rewarming the samples back to 20 °C at a heating rate of 10 °C min^−1^.

Ice crystallization and melting during rapid freezing and rewarming were analyzed to monitor ice crystal formation during cryopreservation. The exothermic peaks were observed in the control and loading treatments during cooling, indicating ice crystallization. The ice melting events were indicated in the control by a negative peak at 0 °C and in the loading treatment through a fluctuating signal (Figure 6). The crystallization formation was not observed after dehydration with PVS3 for 30–90 min or PVS2 for 120–240 min.

## 3. Discussion

American chestnut is an endangered species in North America. Conservation projects for the recovery of American chestnut can benefit tremendously from efficient protocols of mass multiplication and cryopreservation of the available germplasm. Conventional propagation through seeds is limited due to the long and slow reproduction cycle and low seed germination, while vegetative propagation and grafting also produce fewer plants, require specialized grafting skills, and are prone to a high risk of incompatibility [8,9,60,61]. In vitro technology has emerged as a promising tool for conservation of endangered plants due to its ability to rapidly produce true-to-type plants of desired genotypes. In particular, liquid based micropropagation has been shown to be a more efficient method for micropropagation than the semi-solid medium as it enables greater accessibility to nutrients, which subsequently results in a higher multiplication rate with reduced propagation time [62]. Additionally, liquid culture permits automation of the micropropagation in large-scale production systems along with reduced cost of labor and consumables due to the elimination of gelling agents [63,64]. To date, the clonal micropropagation of *Castanea dentata* using shoot explants in liquid culture medium has been understudied. The current study was undertaken to develop an efficient liquid-based micropropagation protocol for American chestnut and assess the efficiency of plant growth regulators in enhancing shoot, root and plantlet development. The temporary immersion culture system developed in this study is also very useful to provide meristematic tissue for ex situ conservation using the droplet vitrification and cryobanking of American chestnut. Together, these protocols provide a basis for similar research studies on micropropagation and cryopreservation for ex situ conservation of diverse chestnut genotypes and many other related endangered tree species. 

The temporary immersion system (TIS) allows for brief, intermittent immersion of plant tissues in the liquid medium along with alternating aeration, which facilitates better propagation than a continuous immersion system (CIS) or a semi-solid culture system (SS) [26]. In the current study, the TIS facilitated better shoot proliferation than the CIS and the SS, which is consistent with observations recorded for other chestnut species. Troch et al. [36] reported the first temporary immersion systems using twin flasks, for propagation of European chestnut plantlets from in vitro culture of shoots. The results showed that apical bud explants produced significantly higher shoot length with more leaves than axillary explants and plantlets grown in the TIS and developed significantly longer shoots than those on a semi-solid medium [36]. Vidal et al. [37] developed a TIS for hybrids of Asian and European chestnuts using axillary shoots and they observed a higher proliferation rate in the liquid medium than in the semi-solid medium for 10 genotypes of chestnut hybrids. Vidal et al. [38] compared the TIS and the CIS for micropropagation of axillary shoots of hybrid chestnuts and demonstrated that both systems generated healthy shoots, but less shoot multiplication was obtained from the CIS, as also recorded in the current study. However, previous reports indicated that hyperhydricity still remained a major issue in chestnut micropropagation using the liquid medium [36,37,38]. In the current study, both the TIS and the CIS showed some symptoms of hyperhydricity, but in the TIS shoot multiplication was generally hyperhydricity free. This may be as a result of the ratio of explants to culture volume in the vessel and the design of our culture vessel. These culture vessels have been developed in our lab and have been found to support a better growth of in vitro cultures for a range of species [65].

Basal salt mixture is one of the important parameters that affect shoot growth and proliferation in American chestnut. WPM is the most commonly used basal medium for shoot proliferation and elongation of American chestnut [12,15,20] and European chestnut [66]. MS is another well-established basal medium that has been widely used in European [67,68,69], hybrid [70,71], Japanese [72], and Chinese chestnut [73]. In the current study, in vitro shoot explants of American chestnut cultured on DKW and MS had better proliferation than shoots grown on WPM. Our results further demonstrated that the application of DKW medium is more efficient than any other salt composition for overall growth of shoots in a liquid-based micropropagation system for American chestnut. A significantly lesser NO3-/NH4+ ratio in the composition of WPM with potentially less available nitrogen for in vitro shoot development may explain these results. Garoosi et al. [74] indicated that ammonium nitrate was the main requirement for optimum in vitro multiplication of *Pistacia vera*. The DKW medium contains relatively higher levels of macronutrients which may be a contributing factor for its positive effect on American chestnut micropropagation.

Plant growth regulators (PGRs) are considered the determinant factors that control plant growth and development in vitro. Cytokinins are commonly added to the basal medium to stimulate shoot formation, promote shoot growth, and facilitate adventitious bud formation [75]. BA, TDZ and zeatin are three commonly used compounds which exhibit strong cytokinin activity for multiplication of in vitro shoot cultures of *Castanea* spp. and BA was found to be the most effective cytokinin for induction and proliferation of shoot explants [68,72,76,77]. Xing et al. [12] successfully proliferated in vitro shoot cultures of American chestnut in semi-solid WPM supplemented with 1 μM BA and 0.5 μM IBA. Soylu and Ertürk [71] compared the effect of various BA concentrations (1–10 mg L^−1^) on shoot multiplication of hybrid chestnut and demonstrated that a high concentration of BA (2–10 mg L^−1^) did not induce shoot growth. The highest shoot multiplication was achieved in a MS (1/2 NO3) medium supplemented with 1 mg L^−1^ BA [71]. Tetsumura and Yamashita [72] compared three cytokinins (BA, TDZ and zeatin) at 5 μM in semi-solid BW medium [78], and observed that the best shoot proliferation in Japanese chestnut occurred with zeatin. However, the current results on American chestnut are different, as zeatin and BA at 4.4 μM induced a similar shoot development response with the DKW medium. These different responses may have arisen due to the use of different basal salt mixture as well as liquid medium in the TIS as compared to semi-solid culture systems. TDZ which was previously reported to be less effective than zeatin and BA in Japanese chestnut, was also found to have little promotive effect on American chestnut in the current study. Tafazoli et al. [67] examined the shoot development of European chestnut on a MS medium containing different levels of BA and TDZ (0.2, 0.5, 1, and 1.5 mg L^−1^); the highest shoot multiplication was obtained with 0.2 mg L^−1^ TDZ and the explants developed calli on 1 mg L^−1^ TDZ. Although calli were also observed at a higher concentration of TDZ than in the present study, shoot elongation and shoot multiplication were significantly reduced in TDZ treatments compared to those with BA. Yang et al. [11] studied in vitro callus induction and shoot initiation of American chestnut by comparing TDZ and zeatin at 0.1, 0.5, 1.5, and 2.0 mg L^−1^ on semi-solid WPM medium. Interestingly, the lowest shoot multiplication was induced with zeatin, and TDZ at lower concentrations resulted in more shoot initiation [11]. A recent study compared BA (1, 2, and 4 mg L^−1^) and TDZ (0.025, 0.05, and 0.1 mg L^−1^) in European chestnut [69] and the results of this study indicated that the maximum multiplication occurred on semi-solid MS (1/2 NO3) medium supplemented with 2 mg L^−1^ BA. Varied responses of cultures grown on different PGRs are likely to be a result of different levels of endogenous PGRs in the tissues and also due to differences in culture conditions, basal media components, and the genotype used in these studies.

Gibberellic acid is known to regulate shoot elongation [79]. Longer shoots are preferred in micropropagation as they provide a higher number of nodes for further proliferation and the ease of handling for periodic subculturing required for maintenance of stock cultures. Currently, limited references are available for GA_3_ application in shoot elongation during in vitro culture of chestnut species. GA_3_ was added to initiation medium [80] and shoot multiplication medium [81] as a supplement for micropropagation of European chestnut. San-Jose et al. [70] found that European chestnut cultures showed no difference in shoot length obtained with or without supplementation with 0.5 mg L^−1^ GA_3_ in semi-solid medium. In the current study, better shoot elongation and longer shoot length were observed in the medium supplemented with 1.0 μM GA_3_ compared to the control (without GA_3_) in the TIS. In vitro responses of explants often vary due to different physiological and genetic backgrounds of the species and cultivars as well as the methods of culture employed.

The inclusion of auxin in the rooting medium facilitates in vitro root initiation and development and the efficiency of rooting is often concentration dependent. Yang et al. [11] demonstrated a superior effect of IBA on root induction compared to NAA at 1.5 and 2.0 mg L^−1^ in a semi-solid culture system for American chestnut. These results are in agreement with the current study in which a significantly higher number of roots was initiated in IBA treatment at 10 μM than those in the NAA-enriched medium (Figure S3). The IBA-supplemented media have been widely used for in vitro rooting of *Castanea* species, including *Castanea crenata*, *C. sativa*, *C. mollissima*, *C. dentata*, as well as hybrid chestnuts [12,67,69,71,73,82,83,84,85]. In the current study, 100% of the shoots developed roots on half-strength DKW medium supplemented with 15 μM IBA and 80% of the rooted shoots were observed in 20 μM IBA supplemented rooting medium (data not shown). Similar to the current study, the addition of 15 μM IBA to half-strength BW medium provided the best rooting percentage (67%) in Japanese chestnut [72]. For European chestnut, the most effective rooting was observed with 4 mg L^−1^ IBA with 81% of explants forming roots [69]. Studies with other chestnut species showed similar rooting responses [76,86,87]. In hybrid chestnut (*C. sativa* × *C. crenata*), root development was stimulated with IBA at 1–3 mg L^−1^ [82,83,84].

In the current study, 100% of the in vitro grown shoots cultured on OASIS^®^ Rootcubes^®^ in liquid TIS developed into rooted plantlets in the medium supplemented with 15 μM IBA. The rooting foams were used as supporting matrices to replace gelling agent in order to mimic a native soil-like environment for in vitro root development, while retaining the advantages of a liquid culture system [88]. The main purpose of the matrix in a liquid culture system is to prevent the asphyxiation of materials that usually occurs during complete immersion in the liquid medium [88]. Rooting foams are ideal platforms for liquid-based culture systems as they reduce the stress of physical handling and separation of rooted plantlets and their transplant to greenhouse occurs with minimal disturbance to the root system. Naylor et al. [89] demonstrated a superior effect of OASIS^®^ foam in liquid-based culture system on root development of micropropagated *Echeveria* compared to the semi-solid culture system. The current study is the first to show the usefulness of OASIS^®^ foam in micropropagation of American chestnut with high success (100%) in initiating root and whole plant development in the TIS.

Plantlets with established roots were acclimatized in the mist bed before transition to the greenhouse. The greenhouse conditions provide relatively lower humidity, higher irradiance, and thus an increased potential for microbial infections which is often stressful to micropropagated plantlets [90]. Micropropagated plantlets were acclimatized in the mist bed under high humidity for two weeks to ease the transition from heterotrophic to autotrophic mode, allowing plants to achieve their inherent potential for photosynthesis in the ex vitro environment. American chestnut plantlets showed 100% survival in the mist bed and 82% survival after one month of establishment in the greenhouse. Similar efficiencies of greenhouse transplant have been reported for other chestnut species including Japanese chestnut, in which 85% survival was observed after 30 days of growth in the greenhouse [72].

A droplet-vitrification cryopreservation protocol was also developed in the current study for in vitro grown shoot tips of American chestnut. An effective droplet-vitrification cryopreservation has not been reported in *Castanea* species, but the technique has been widely applied successfully to numerous plant species, such as banana [91], rose [92], taro [50], apple [48,93], cherry plum [94], lily [53], blueberry [95], and shallot [96]. With regards to chestnut species, different cryopreservation protocols have been reported for European chestnut. Jorquera et al. [44] reported cryopreservation of European chestnut using a two-step vitrification methodology. With this protocol, micropropagated *Castanea sativa* shoot tips were cryopreserved with 42% shoot recovery from terminal buds in clone 818 and 37.5% in clone 12 [44]. Similarly, Vidal et al. [46] reported cryopreservation of five clones of *Castanea sativa* (clone 12, 812, and 818 of juvenile tissue origin, and LA3 and PR5 of mature tissue origin) using the vitrification procedure. The overall shoot recovery obtained from cryopreserved apical meristems following vitrification was 53.3% (clone 818), 37.5% (clone 12), 53.2% (clone 812), 54.4% (clone LA3), and 42.5% (clone PR5) [46]. Further, Vidal et al. [97] described a vitrification-based cryopreservation procedure that was applied to 46 genotypes of in vitro grown European chestnut. The shoot recovery efficiency ranged from 0% to 53%, indicating a significant genotype-specific response of *Castanea sativa* [97].

The preculture treatment of explant materials is essential to obtain a high recovery rate of cryopreserved samples. In the present study, the stepwise increase of sucrose concentration during preculture treatments produced the highest recovery of shoot tips after cryopreservation. Shoot tips of American chestnut were precultured stepwise for 96 h in sucrose-enriched solution with concentrations ranging from 0.25 to 1.0 M, which significantly shortened the preculture duration. The progressive increase of sucrose concentration during preculture treatments was found to improve the survival and regeneration of numerous species such as *Corrigin grevillea* [98], sugar beet [99], black locust [100], potato [101], and grapevine [102]. Jorquera et al. [44] indicated that shoot tips of European chestnut provided a sufficient recovery rate when precultured in sucrose concentrations between 0.1 to 0.4 M for two days at 4 °C, while shoot tips precultured in higher sucrose concentration (0.7 M) showed a significantly decreased recovery (7.4%). The present study also confirmed that preculturing shoot tips of American chestnut on 0.5 M sucrose for 48–72 h at room temperature allowed good survival and regeneration, while shoot tips precultured with 1.0 M sucrose failed to survive. A two-step cold hardening protocol has been commonly used in cryopreserved shoot tips of European chestnut to induce tolerance of plant material to dehydration and subsequent freezing in liquid nitrogen [44,45,46,97]. According to these studies, shoot tips of *C. sativa* required a minimum of two to three weeks of cold hardening before cryopreservation, and subsequently resulted in longer period of cryopreservation process. The preculture treatment in the current study with American chestnut significantly reduced the duration of preculture procedure with no need for cold hardening, while maintaining regeneration ability of the shoot tips.

Following preculture, samples are loaded with a cryoprotectant solution, also referred to as a loading solution for osmoprotection, in order to prevent possible damage during dehydration [103]. Cryopreserved shoot tips are commonly pre-treated with a loading solution consisting of 2 M glycerol and 0.4 M sucrose for 20 min, as this has proven to be efficient in enhancing tolerance to subsequent PVS exposure [103,104,105]. The present study followed the same composition and duration of loading treatment to attain shoot recovery by droplet vitrification protocols. Shoot tips of European chestnut were also treated similarly with the loading solution prior to dehydration [44,45,46,97]. 

Dehydration with concentrated cryoprotectant solution is the determinant step in vitrification-based cryopreservation procedures. Samples are required to be exposed to a plant vitrification solution for dehydration before immersion in liquid nitrogen. The plant vitrification solutions, especially PVS2 and PVS3, can replace cellular water in order to protect the cells during cryostorage [106,107]. In the current study, both PVS2 and PVS3 were successfully used during cryopreservation of in vitro shoot tips of American chestnut. Since dehydration is the step that causes the major damage to cryopreserved samples, optimization of exposure duration and the choice of PVS are critical for achieving high survival and regeneration [103]. The shoot tips exposed to PVS2 for 150 min showed an optimal regeneration of 48% through droplet-vitrification cryopreservation in our experiments. Similar results were obtained in European chestnut in which shoot tips exposed to PVS2 for 120 min at 0 °C showed maximum recovery (45.6%) in vitrification cryopreservation [44,46]. The composition of PVS2 includes ethylene glycol and DMSO that are osmotic cryoprotectants with strong ability to rapidly penetrate cell walls and membranes [106]. The exposure to PVS2 should be carried out at lower temperatures, usually with ice, to reduce toxicity and damage [105]. PVS3 is an alternative cryoprotective solution for vitrification-based cryopreservation. In the current study, PVS3 was used for dehydration during cryopreservation of chestnut species and 30 min exposure to PVS3 produced optimal regeneration of 55%. The application of PVS3 has not been studied in other *Castanea* species yet, but the effect of PVS3 has already been confirmed in vitrification-based cryopreservation of a number of species, such as apple [108], garlic [43], asparagus [104], pineapple [109], and chrysanthemum [110]. In the present study, although PVS2 and PVS3 both stimulated sufficient regeneration of cryopreserved shoot tips in droplet-vitrification, the PVS3 appeared to be a better solution as it requires a reduced dehydration period. In addition, PVS3 is less toxic than PVS2 due to the absence of ethylene glycol and DMSO, which results in easier operation of PVS3 without the need for ice-cooling during the dehydration process. Kim et al. [110] also showed a superior effect of PVS3 compared to PVS2 in cryopreserved shoot tips of garlic and chrysanthemum during droplet vitrification. Both garlic and chrysanthemum were sensitive to chemical toxicity of cryoprotectant solutions and cryopreserved shoot tips of both species showed a higher recovery by PVS3 dehydration compared to PVS2 [110].

Post-culture after cryostorage is another important factor that directly influences the recovery of cryopreserved samples. Cryopreserved shoot tips of American chestnut were post-cultured on DKW supplemented with 2.2 μM BA and 1.0 μM GA_3_ in the dark for five days, followed by dim light, and subsequently normal light. The addition of BA in the post culture medium significantly improved the survival of cryopreserved shoot tips in grape and citrus [111]. In European chestnut, shoot tips following vitrification cryopreservation were post-thawed on a recovery medium [112] supplemented with 0.5 mg L^−1^ BA and 0.5 mg L^−1^ IAA [44,45,46,97].

The differential scanning calorimetry analysis was done by exposing shoot tips of American chestnut, treated with various cryoprotective steps, to rapid freezing and rewarming cycle in order to detect the glass transition. Ice nucleation signal was observed during the freezing and rewarming as crystallization and melting of the non-vitrified cellular water occurred in the non-cryoprotected fresh shoot tips only. The disappearance of the ice nucleation indicated a proper dehydration of shoot tips with PVS2 and PVS3 without the formation of ice during the rapid cooling and rewarming. Successful cryopreservation through droplet-vitrification protocols has been monitored by a differential scanning calorimeter in a number of species, such as *Lomandra sonderi* [113], *Rubia akane* [114], *Atractylodes macrocephala* [115], and *Betula lenta* [55]. These thermogram results confirmed that samples after a series of cryoprotective procedures were ready for cryostorage at super-cooling temperatures. 

## 4. Materials and Methods

### 4.1. Micropropagation

#### 4.1.1. Initiation and Establishment of Shoot Cultures

Shoot buds were collected from three-year-old plants (Verbinnen’s Nursery, ON, Canada) grown in greenhouse conditions for shoot initiation in vitro. Buds were surface sterilized with 15% (*v/v*) commercial bleach (Clorox^®^; The Clorox Company, Oakland, CA, USA; 5.4% sodium hypochlorite) with two drops of Tween-20 (Sigma Aldrich, St. Louis, MO, USA) for 20 min. They were then rinsed with autoclaved deionized water, four times, 3 min each wash. Shoot buds after sterilization were cultured in an upright position in test tubes on a semi-solid medium containing ingredients of DKW salt mixture with vitamins [116] (PhytoTechnology Laboratories^®^, Lenexa, KN, USA) supplemented with 3% sucrose, and 2.2 g L^−1^ Phytagel™ (Sigma Aldrich, Oakville, ON, Canada). The pH of the medium was adjusted to 5.75 prior to autoclaving for 20 min at 121 °C and 118 kPa. After three weeks of culture in the test tubes, the explants were transferred to Magenta GA7 vessels (Sigma Aldrich, Oakville, ON, Canada) containing the same medium for further shoot development. Shoot growth of primary explants was observed for six weeks of culture. Subsequently, shoots were excised and sub-cultured for further growth onto a fresh DKW medium of the same composition as described above. 

#### 4.1.2. Shoot Proliferation

Nodal explants obtained from in vitro grown shoots were sub-cultured in culture vessels [65] each containing 50 mL of liquid DKW shoot multiplication medium. The vessel was 85 mm wide, 235 mm long, and 80 mm high with a lid that was 85 mm wide, 235 mm long, and 12 mm high. The pH of the medium was adjusted to 5.7 followed by autoclaving for 20 min at 121°C and 118 kPa. All culture vessels were placed on ‘Cultureshift’, a rocker-based temporary immersion system (VRE Systems, ON, Canada) set at 22 ± 2 °C with a 16-h photoperiod provided by fluorescent tubes (50 μmol m^−^^2^ s^−^^1^; EiKO^®^, Kansas City, KS, USA). The mechanical movement of the rocker system allowed vessels to tilt at 30 degrees on both sides so that the shoot explants can be alternately immersed in the medium and exposed to aeration for 30 s during each cycle.

Three different basal salt mixtures with vitamins (obtained from PhytoTechnology Laboratories^®^, Shawnee Mission, KS, USA) were compared for their efficiency in stimulating shoot proliferation: DKW (Driver and Kuniyuki Walnut Medium); MS (Murashige and Skoog Medium) [117]; and WPM (Lloyd and McCown Woody Plant Medium) [118]. Each medium was supplemented with 3% sucrose, 2.2 µM 6-benzylaminopurine and 1.0 µM gibberellic acid (BA and GA_3_; PhytoTechnology Laboratories^®^). Each treatment was replicated three times with eight to ten explants per replicate. Observations were taken for the number of shoots formed on each explant and the shoot length after three weeks of growth.

The optimal cytokinin treatment was determined by subculturing shoot explants in medium with either BA, Zeatin (Caisson Laboratories Inc., Smithfield, UT, USA), or thidiazuron (TDZ; Caisson Laboratories Inc.) at 1.1, 2.2, or 4.4 µM as well as the control without the addition of any cytokinin. All treatments contained 3% sucrose, DKW basal salt mixture with vitamins, and 1.0 µM GA_3_. Each treatment was replicated three times with eight to ten explants per replicate. Observations were taken for the number of shoots formed on each explant and the shoot length after six weeks of growth.

The effect of gibberellic acid was examined through the addition of GA_3_ at 0.0 (control), 0.5, 1.0, or 2.0 µM concentrations in the medium containing DKW basal salt mixture with vitamins, 3% sucrose, and 2.2 µM BA. Each treatment was replicated three times with eight to ten explants per replicate. Number of long shoots (≥0.5 cm), longest shoot length, and total number of shoots were determined for comparison of the effects of different GA_3_ concentrations after six weeks of growth.

#### 4.1.3. Comparison of Semi-Solid System (SS), Continuous Immersion System (CIS), and Temporary Immersion System (TIS) for In vitro Growth 

To determine the optimal growth system for shoot proliferation, shoot explants were cultured in three culture systems: (1) liquid temporary immersion system (TIS); (2) liquid continuous immersion system (CIS); (3) semi-solid culture system (SS). Each culture system contained the DKW basal salt mixture with vitamins, 3% sucrose, 2.2 μM BA, and 1.0 μM GA_3_. The phytagel (2.2 gL^−^^1^) was used only in the semi-solid culture system. Explants in all treatments were cultured in vessels placed on the rocker system. The TIS treatments were placed on the mobile shelves to achieve temporary immersion of explants, while the CIS treatment and the semi-solid treatment were put on immobile shelves of the rocker. The immobile shelf kept the shoot explants continuously immersed in the stationary liquid culture medium for the CIS treatment. Each treatment was replicated three times with eight to ten explants per replicate. Observations were taken for the number of shoots formed on each explant and the longest shoot length after three weeks of growth.

#### 4.1.4. Rooting and Plantlet Development

Microshoots obtained from cultures in shoot proliferation experiments were treated with either Indole-3-butyric acid (IBA; Sigma Aldrich, Oakville, ON, Canada) or 1-Naphthaleneacetic acid (NAA; PhytoTechnology Laboratories^®^) for root induction. Shoots approximately 30–40 mm in length were excised, inserted vertically in sterile rooting foam (OASIS^®^ Grower Solutions Rootcubes^®^ Wedge^®^), and cultured in vessels kept on the TIS. The rooting medium in each treatment contained 3% sucrose, half-strength DKW basal medium with vitamins as well as supplemented with either IBA or NAA at 0, 2.5, 5.0, and 10 μM concentrations. Each treatment was replicated four times with nine shoots per replicate. Observations were recorded for the number of roots on each explant. Since efficient root development was observed in IBA treatments compared to NAA, the optimal IBA concentration for root growth was determined by culturing shoots in liquid rooting culture system as described earlier with IBA at 5, 10, 15, 20 μM concentrations. Each treatment was replicated four times with nine shoots per replicate. The percentages of rooted shoots, the number of roots and the longest root length were measured after three weeks of culture. Root induction and development were also examined in the semi-solid medium as compared to the shoots rooted in the liquid TIS with Rootcubes^®^. Observations were taken for the percentage of rooted explant in the semi-solid medium after five weeks of growth.

#### 4.1.5. Greenhouse Acclimatization

Rooted plantlets were transplanted into cell trays (4 × 10) filled with Sunshine professional growing media (Sun Gro Horticulture Canada Ltd., Brantford, ON, Canada). Plantlets were initially acclimatized in the mist bed (24 °C for 16 h light and 20 °C for 8 h darkness, with 85% humidity) for two weeks in order to allow adaptation to environmental changes and later transferred in the greenhouse (24 °C for 16 h light and 20 °C for 8 h darkness, light intensity at 110 μmolm^−2^s^−^^1^). The survival (surviving plants/total rooted plants) was recorded after four weeks of growth in the greenhouse.

### 4.2. Cryopreservation

The stock plants were sub-cultured in an optimized liquid shoot multiplication medium (DKW basal medium supplemented with 3% sucrose, 2.2 μM BA, and 1.0 μM GA_3_). Shoot tip explants (1.0–1.5 mm long) containing five to six leaf primordia were excised from 4-week-old stock cultures (Figure 1F) and stabilized overnight in the dark on semi-solid shoot multiplication medium for establishing the droplet-vitrification method for cryopreservation.

The effect of sucrose pretreatment was determined by preculturing shoot tips in different sucrose-enriched solutions (0, 0.25, 0.5, 1.0 M) for various durations (0, 24, 48, 72, 96 h). In another preculture treatment, shoot tips were precultured stepwise in varying concentrations (0.25–1.0 M) of sucrose for 96 h. Shoot tips were exposed to 0.25 M sucrose for the first 24 h; then to 0.5 M sucrose for 24 h; 0.75 M sucrose for 24 h; and 1.0 M sucrose for 24 h. The survival and regeneration percentage of shoot tips in stepwise preculture treatment was compared to the 0.5 M sucrose preculture treatment to determine the optimal pretreatment solution. 

Following the preculture step, shoot tips were loaded in a loading solution (LS) comprising 2 M glycerol and 0.4 M sucrose for 20 min. After loading, shoot tips were dehydrated with a plant vitrification solution (PVS2 or PVS3). The vitrification solution PVS2 contained MS [117] (Murashige and Skoog, 1962) basal ingredients supplemented with 30% glycerol (*w*/*v*), 15% ethylene glycol (*w*/*v*), 15% dimethyl sulfoxide (DMSO; *w*/*v*) and 0.4 M sucrose at pH 5.7 [119]. The vitrification solution PVS3 consisted of 50% glycerol (*w*/*v*) and 50% sucrose (*w*/*v*) in liquid MS medium (pH 5.7) [104]. Shoot tips were treated with ice cooled PVS2 for 90, 120, 150, 180, 210, or 240 min or exposed to PVS3 at room temperature for 0, 30, 60, 90, or 120 min. Each treatment contained three replicates and each replicate included ten shoot tips placed in a Magenta™ B-cap glass jar (Sigma Aldrich, Oakville, ON, Canada) containing 20 mL medium. The effect of exposure duration to both PVS2 and PVS3 was determined by regeneration efficiency after cryopreservation. All explants from each treatment were placed in a separate Magenta™ B-cap glass jar (Sigma Aldrich, Oakville, ON, Canada) on a shaker at 100 rpm (Max™ 2000 Benchtop Orbital Shaker, Thermo Scientific™) for proper agitation. Each treatment contained 30 shoot tips and each glass jar had 10 shoot tips.

Following dehydration, five shoot tips were transferred onto an aluminum foil strip (1 × 4 cm) with droplets (about 3–4 µL) of PVS2 or PVS3 (Figure 1G), followed by direct immersion into liquid nitrogen (LN) (+LN). After 1 h in LN, the frozen foils with shoot tips were removed from LN and immediately plunged into an unloading solution (1.2 M sucrose), warmed up in a hot water bath (40 °C) for a rapid rewarming for 30–60 s, and subsequently transferred to the unloading solution at room temperature for 20 min. Meristems without treatment in LN (−LN) were directly exposed to the unloading solution after dehydration and considered as the control. Both cryopreserved shoot tips (+LN) and the treated control (−LN) were post-cultured on semi-solid medium in Petri dishes (Fisherbrand™ Petri Dishes with Clear Lid; 60 mm × 15 mm) containing DKW medium with Vitamins (PhytoTechnology Laboratories^®^), 3% sucrose, 2.2 μM BA, 1.0 μM GA_3,_ and 2.2 g L^−^^1^ phytagel^®^ (Sigma Aldrich, Oakville, ON, Canada) at pH 5.7. For optimizing regeneration, thawed shoot tips treated with PVS3 for 30 min were placed on the sterile filter paper, which was soaked with two types of the post-culture solution: a) DKW supplemented with 3% sucrose, 2.2 µM BA, and 1.0 µM GA_3_, pH 5.7; and b) DKW supplemented with 3% sucrose, 2.2 µM BA, and 2.9 µM IAA, pH 5.7. The solutions were refreshed every ten days. Each Petri dish contained 10 shoot tips and there were three Petri dishes per treatment. These cultures were kept in the dark for five days, dim light condition of 5 μmolm^−2^s^−^^1^ for five days, and then transferred to normal culture room light (40 μmolm^−^^2^s^−1^) for regeneration. Regeneration was determined as the percentage of cryopreserved shoot tips showing green color and growth under microscope after four weeks of culture in post culture medium. Shoot regrowth was calculated as the percentage of shoot tips that developed into normal shoots (≥ 5 mm in length) at 8-weeks post-culture. Shoot tips precultured in different treatments were plated on the post-culture medium without cryopreservation to determine the survival of the shoot tips after pretreatments. The survival of precultured explants was defined as the percentage of shoot tips showing yellowish green color after four weeks of recovery on post-culture medium.

#### Differential Scanning Calorimetry Analysis

A thermo-analytical technique, Differential Scanning Calorimetry (DSC; Mettler Toledo, Leicester, UK), was used to determine the thermal phase transitions in shoot tips of American chestnut during cryopreservation. Samples were weighted and sealed in standard aluminum pans and placed in the DSC. The DSC was cooled down from 20 °C to −80 °C with a cooling rate of −10 °C min^−^^1^ and held isothermally for 5 min prior to rewarming back to 20 °C at a heating rate of 10 °C min^−^^1^. Tested samples included: (I) Control: Fresh shoot tips excised from stock culture without any cryopreserved treatment; (II) Loading: Shoot tips precultured in stepwise concentration of sucrose for four days followed by loading solution for 20 min; (III) PVS3: Shoot tips precultured in stepwise concentration of sucrose for four days followed by loading solution for 20 min, subsequently dehydrated in PVS3 for 30, 60, 90, and 120 min; and (IV) PVS2: Shoot tips precultured with stepwise concentration of sucrose for four days followed by loading solution for 20 min, subsequently dehydrated in PVS2 for 120, 180, 240 min. Each treatment was replicated twice. Curves were analyzed by STARe thermal analysis software (Mettler Toledo, Leicester, UK).

### 4.3. Statistical Analysis

All the experiments were replicated twice and conducted in a complete randomized design. Treatment groups included a minimum of three biological replicates with eight to ten explants per culture vessel. Data were analyzed using a generalized linear mixed model (GLIMMIX) for all experiments. Normality was tested using Shapiro–Wilk’s test of normality. Means were compared through Tukey–Kramer Honestly Significant Difference (HSD) test with α = 0.05. All statistical data were presented as means ± standard error; different letters demonstrate significant differences. The SAS system Proc Glimmix (SAS University Edition, SAS Institute, Cary, NC, USA) was used for all statistical computations.

## 5. Conclusions

In summary, an in vitro technology using liquid-based temporary immersion system (TIS) was optimized for micropropagation of American chestnut. The in vitro shoot tip explants were multiplied with optimization of basal medium and plant growth regulators and rooted plantlets were successfully transferred to the greenhouse. The micropropagation protocol developed provides a consistent supply of large numbers of regenerable shoot tips for mass multiplication and cryopreservation using droplet vitrification for germplasm preservation. This study demonstrates and reaffirms the usefulness of in vitro technologies for the propagation and long-term conservation of endangered species such as American chestnut. As such, the findings of this study may be useful for developing in vitro methods of conservation for other recalcitrant woody plant species.

## Figures and Tables

**Table 1:** The regeneration percentages of shoot tips after continuous and stepwise preculture treatments. Shoot tips were precultured in 0.5 M sucrose for continuous 24–96 h and stepwise concentration of sucrose (0.25 to 1.0 M) for 96 h, followed by loading and dehydration through PVS3 exposure for 30 min. Shoot tips with (+LN) and without (−LN) cryopreservation were unloaded and plated on post-culture medium. The effect on regeneration was observed after four weeks of growth on post-culture medium. Data represent means ± standard errors. Means followed by same letters are not significantly different at *p* = 0.05 according to Tukey–Kramer HSD test.

Continuous Preculture	+LN (%)	−LN (%)
0.5 M (24 h)	00.0 ± 4.6 c	86.7 ± 8.3
0.5 M (48 h)	38.3 ± 4.6 ab	80.0 ± 8.3
0.5 M (72 h)	40.0 ± 4.6 a	76.7 ± 8.3
0.5 M (96 h)	20.0 ± 4.6 b	70.0 ± 8.3
Stepwise Preculture		
0.25–1.0 M (96 h)	55.0 ± 4.6 a	76.7 ± 8.3

## Data Availability

Not applicable.

## Supplementary Materials

The following supporting information can be downloaded at: https://www.mdpi.com/article/10.3390/plants11030464/s1, Figure S1: The effect of basal salt mixtures on shoot proliferation. Number of shoots (A) and longest shoot length (B) were recorded for three different basal salt mixtures (DKW, MS, and WPM). Error bars represent the mean ± standard error. Means followed by the different letter are significantly different according to Tukey–Kramer HSD test (α = 0.05). Figure S2: Evaluation of various levels of Gibberellic acid (0, 0.5, 1.0, and 2.0 μM) on number of long shoots (≥0.5 cm) (A); longest shoot length (B); number of dwarf shoots (<0.5 cm) (C); and total number of shoots (D) of in vitro shoot culture of American chestnut. Error bars represent the mean ± standard error. Means followed by the different letter are significantly different according to Tukey–Kramer HSD test (α = 0.05). Figure S3: The effect of auxins (NAA and IBA) at 0, 2.5, 5.0, and 10 μM on number of primary roots during in vitro root development. Error bars represent the mean ± standard error. Means followed by the different letter are significantly different according to Tukey–Kramer HSD test (α = 0.05).
