# Supplementary material for: In Vitro Technologies for American Chestnut (Castanea dentata (Marshall) Borkh) Conservation
Source: Plants (Basel). 2022 Feb 8;11(3):464. doi: 10.3390/plants11030464 (PMC8840129; doi:10.3390/plants11030464)
Supplement: Supplementary file 1 [file plants-11-00464-s001.zip › plants-1580920-supplementary.pdf]

In Vitro Technologies for American Chestnut (*Castanea dentata* (Marshall) Borkh. Conservation

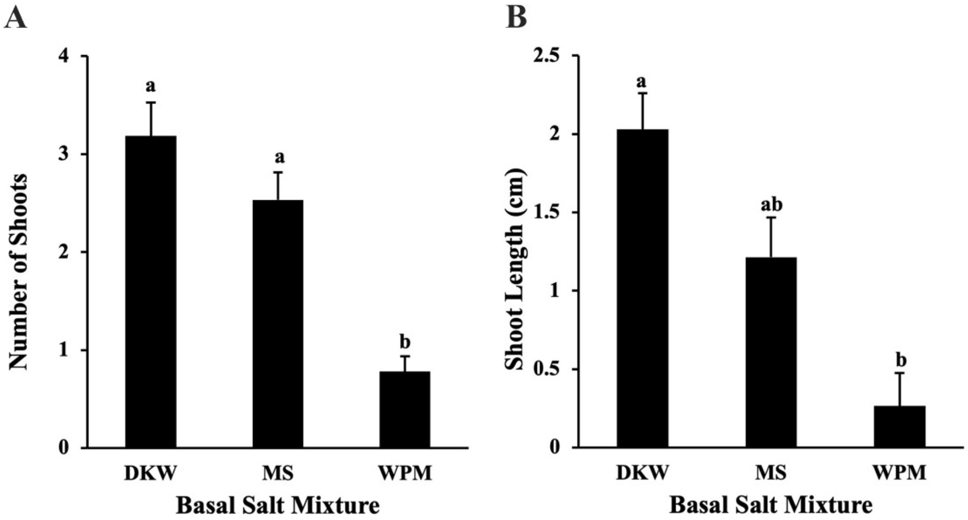

Figure S1: The effect of basal salt mixtures on shoot proliferation. Number of shoots (A) and longest shoot length (B) were recorded for three different basal salt mixtures (DKW, MS, and WPM). Error bars represent the mean ± standard error. Means followed by the different letter are significantly different according to Tukey–Kramer HSD test (α = 0.05).

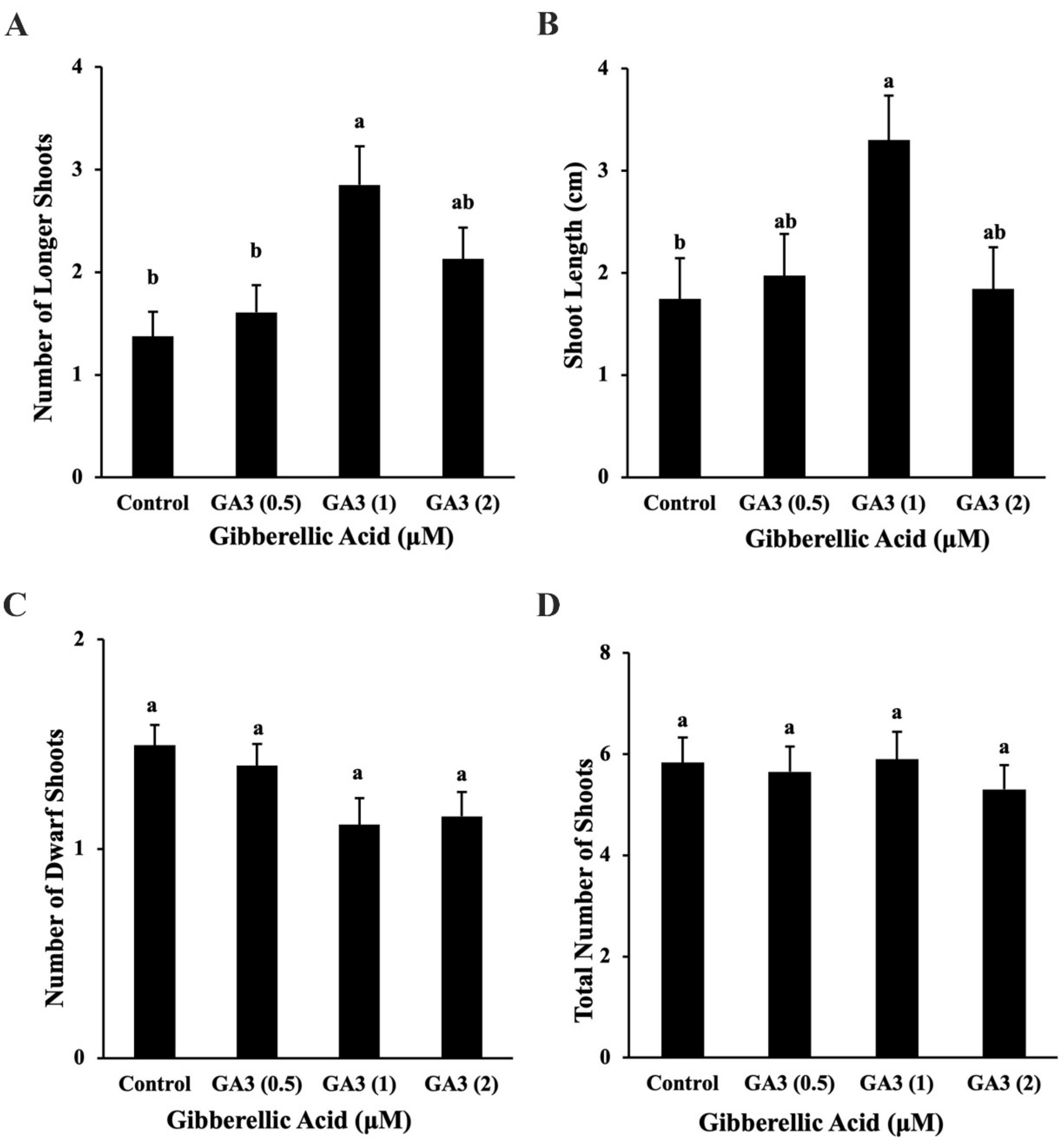

Figure S2: Evaluation of various levels of Gibberellic acid (0, 0.5, 1.0, and 2.0 µM) on number of long shoots (≥ 0.5 cm) (A); longest shoot length (B); number of dwarf shoots (<0.5 cm) (C); and total number of shoots (D) of *in vitro* shoot culture of American chestnut. Error bars represent the mean ± standard error. Means followed by the different letter are significantly different according to Tukey–Kramer HSD test (α = 0.05).

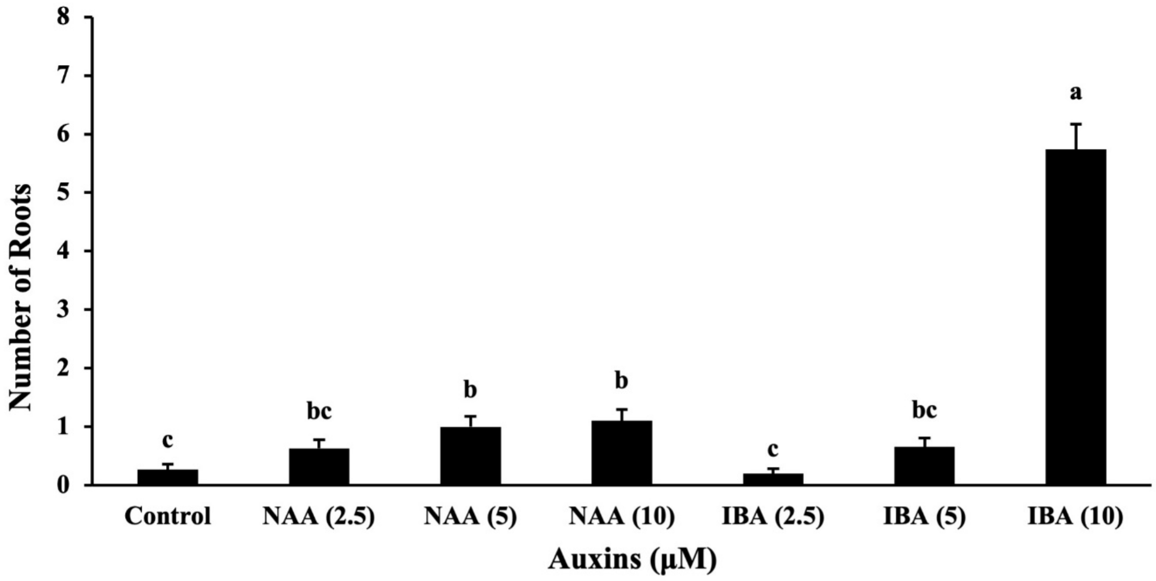

Figure S3: The effect of auxins (NAA and IBA) at 0, 2.5, 5.0, and 10 μM on number of primary roots during *in vitro* root development. Error bars represent the mean ± standard error. Means followed by the different letter are significantly different according to Tukey–Kramer HSD test (α = 0.05)
